# Non-thermal resistive switching in Mott insulator nanowires

**DOI:** 10.1038/s41467-020-16752-1

**Published:** 2020-06-12

**Authors:** Yoav Kalcheim, Alberto Camjayi, Javier del Valle, Pavel Salev, Marcelo Rozenberg, Ivan K. Schuller

**Affiliations:** 1 Department of Physics and Center for Advanced Nanoscience, University of California-San Diego, La Jolla, CA 92093 USA; 2 Departamento de Física, FCEyN, UBA and IFIBA, Conicet, Pabellón 1, Ciudad Universitaria, Buenos Aires, 1428 CABA Argentina; 3 Université Paris-Saclay, CNRS, Laboratoire de Physique des Solides, 91405 Orsay, France

**Keywords:** Electrical and electronic engineering, Electronic properties and materials, Phase transitions and critical phenomena, Electronic and spintronic devices

## Abstract

Resistive switching can be achieved in a Mott insulator by applying current/voltage, which triggers an insulator-metal transition (IMT). This phenomenon is key for understanding IMT physics and developing novel memory elements and brain-inspired technology. Despite this, the roles of electric field and Joule heating in the switching process remain controversial. Using nanowires of two archetypal Mott insulators—VO_2_ and V_2_O_3_ we unequivocally show that a purely non-thermal electrical IMT can occur in both materials. The mechanism behind this effect is identified as field-assisted carrier generation leading to a doping driven IMT. This effect can be controlled by similar means in both VO_2_ and V_2_O_3_, suggesting that the proposed mechanism is generally applicable to Mott insulators. The energy consumption associated with the non-thermal IMT is extremely low, rivaling that of state-of-the-art electronics and biological neurons. These findings pave the way towards highly energy-efficient applications of Mott insulators.

## Abstract (web summary)

Despite intensive research on the electrically driven insulator-to-metal transition, this phenomenon is not well understood. Using quasi 1D nanowires of two Mott insulators, the authors reveal the central role of defects in enabling a non-thermal doping driven insulator-to metal transition.

## Introduction

The insulator–metal transition (IMT) in strongly correlated materials is a phase transition characterized by drastic changes in electrical properties, which may be accompanied by structural and magnetic transitions. The richness of this phenomenon has made it one of the most studied topics in condensed matter physics. The properties of IMT materials and their sensitivity to external stimuli make them promising for applications, such as memory, selectors for ReRAM, optical switches, and emulating brain functionalities^1–17^. Another advantage of these systems is that the IMT can be triggered by various perturbations, such as optical pumping or changes in temperature, pressure, strain, and chemical doping. The most practical way of inducing the IMT in electronic devices is by applying electrical current or voltage^18,19^. Such devices can be very fast^6,20^ and highly scalable^17^.

The mechanism behind the electrically triggered IMT is currently under heavy debate in the scientific community. In materials where the IMT takes place as a function of temperature, such as VO_2_ and V_2_O_3_, Joule heating due to current flow is an obvious candidate for triggering the transition. It has been argued, however, that the electric field applied in this process may induce the IMT non-thermally, without heating the material to its IMT temperature (*T*_IMT_)^21–27^. Despite many studies, the switching mechanism is not yet understood. Even for the same materials, in some cases Joule heating is invoked, while in others non-thermal effects of the electric field may play an important role^20–36^. In a few studies, field-assisted carrier generation has been invoked as a possible driving mechanism for this non-thermal IMT, but no clear evidence linking the two phenomena was shown.

If non-thermal switching is indeed possible, it may have significant advantages for energy consumption, mechanical stability, and time scales as compared to the Joule heating scenario. The greatest challenge in distinguishing between the effect of Joule heating and electric field is in determining the spatial distribution of current and temperature in a device. Because of the low resistivity of metallic domains compared to insulating ones, current flow can become highly non-uniform, resulting in inhomogeneous Joule heating and electric field profiles. For example, in quasi-2D VO_*x*_ thin films, the current flow is filamentary which can dramatically alter the local temperature distribution^19,37^. This hinders the determination of the mechanism behind the electrically triggered IMT, since it is unclear whether the transition occurs because the IMT temperature is locally attained under an applied field.

In this work, we study the electrically triggered IMT in quasi-1D nanowires of two archetypal Mott insulators exhibiting an IMT: VO_2_ and V_2_O_3_. Through accurate temperature calibration, we find that in both materials thermal and purely non-thermal IMTs are possible, thereby settling apparent contradictions regarding the origin of the field-induced IMT. We clearly identify the crucial role of defects in determining the switching mechanism and demonstrate a means of controlling it. Our findings show that defects enhance the efficiency of field-assisted carrier generation, thereby enabling a non-thermal doping driven IMT. The similarity between the switching properties in VO_2_ and V_2_O_3_, despite their different ground states, suggests that this mechanism is applicable to many other Mott insulators. By avoiding heat dissipation and its associated dynamics, the energy consumption and operation timescales of IMT-based devices may be greatly reduced.

## Results

**Fig. 1:**
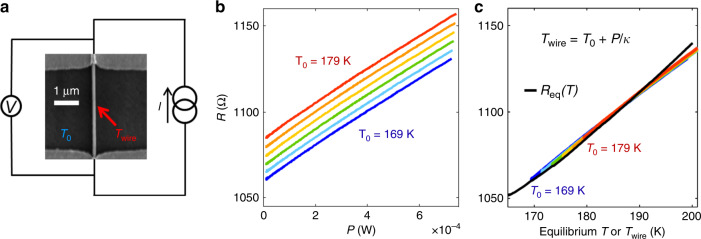
Determination of temperature change due to applied power in VO*x* nanowires. **a** SEM image of a V_2_O_3_ nanowire contacted by two Ti/Au pads (top and bottom) and schematic of the measurement setup. *T*_0_ and *T*_wire_ are the temperatures of the substrate and the nanowire, respectively, which differ due to the applied power. **b** Resistance vs. power curves measured above the IMT temperature in the fully metallic state of a V_2_O_3_ nanowire at *T*_0_ between 169 and 179 K (blue to red). **c** Resistance vs. *T*_wire_ (colored lines) derived from **b** using Eq. (2) with a single fitting parameter *κ* = 24 μW K^−1^ for all curves (colored). The black curve shows the resistance vs. stage temperature at equilibrium, *R*_eq_(*T*). The horizontal *T* axis refers both to the stage temperature during the equilibrium (low power) measurement and *T*_wire_.

We employed advanced fabrication techniques to prepare high-quality nanowires of VO_2_ and V_2_O_3_ (see scanning electron micrograph in Fig. 1a and “Methods” section). Our samples show extremely sharp 4–6 orders of magnitude resistance change across the IMT (Supplementary Fig. 1a, b). The nanowire widths (~100 nm) are comparable to the size of insulator/metal domains, as found in a previous study^38^, and further supported by resistor network simulations (Supplementary Note 1 and Fig. 1c–e)^1^. The advantage of using such quasi-1D structures is that spatial confinement suppresses filament formation during the electrically triggered IMT. This greatly simplifies data interpretation and facilitates a clear distinction between thermal and electric field effects. Another advantage, as compared to the nano-gap geometry, is that nanowires are robust to power surges and thermal runaway upon nucleation of metallic domains because the remaining non-switched insulating domains act as current limiting series resistors (see Supplementary Note 3 and footnote 1). Considering homogeneous resistivity and current distributions in the nanowire, the effect of Joule heating on the nanowire temperature (*T*_wire_) can be quantitatively determined from a simple heat equation:

1 $$C\frac{{{\mathrm{d}}T_{{\mathrm{wire}}}}}{{{\mathrm{d}}t}} = IV + \kappa \left( {T_0 - T_{{\mathrm{wire}}}} \right)$$

where *I* and *V* are the current and voltage applied to the nanowire, *C* is its heat capacitance, *T*_0_ is the (measured) substrate temperature and *κ* is the thermal coupling constant between the substrate and the nanowire. Equation (1) is strictly valid when resistivity, power, and temperature variations along the nanowire can be neglected. This is the case in the single-phase homogeneous metallic and insulating states, above and below the IMT hysteresis. As will be shown subsequently, empirically, this equation still provides a very good approximation even when the two states coexist. In steady state $\frac{{{\mathrm{d}}T_{{\mathrm{wire}}}}}{{{\mathrm{d}}t}} = 0$ and thus

2 $$T_{{\mathrm{wire}}} = P/\kappa + T_0$$

where the power is *P* = *IV*. To measure *κ* we performed a series of voltage vs. current measurements, *V*(*I*), at different *T*_0_ corresponding to the metallic state. From this we derived resistance vs. power curves—*R*(*P*), which show a trend of increasing *R* with *P* (see Fig. 1b). A similar trend is observed in resistance vs. substrate temperature curves which were measured at thermal equilibrium [*R*_eq_(*T*)] by applying minimal current/power to the sample and changing the stage temperature. Using Eq. (2) to relate *P* and *T*_wire_, we successfully collapsed all the *R*(*P*) curves onto the *R*_eq_(*T*), as shown in Fig. 1c. We note that all curves were collapsed using a single fitting parameter *κ*. For the nanowires discussed in this work, *κ* ranged between 21 and 24 μW K^−1^ for the V_2_O_3_ devices and ~45 μW K^−1^ for the VO_2_ device (see Supplementary Note 2 for comparison of *κ* values obtained in previous work and footnote 1). Knowing these thermal coupling constants allows us to accurately quantify Joule heating and enables a reliable estimate of *T*_wire_ from the measured parameters *T*_0_ and *P*.

**Fig. 2:**
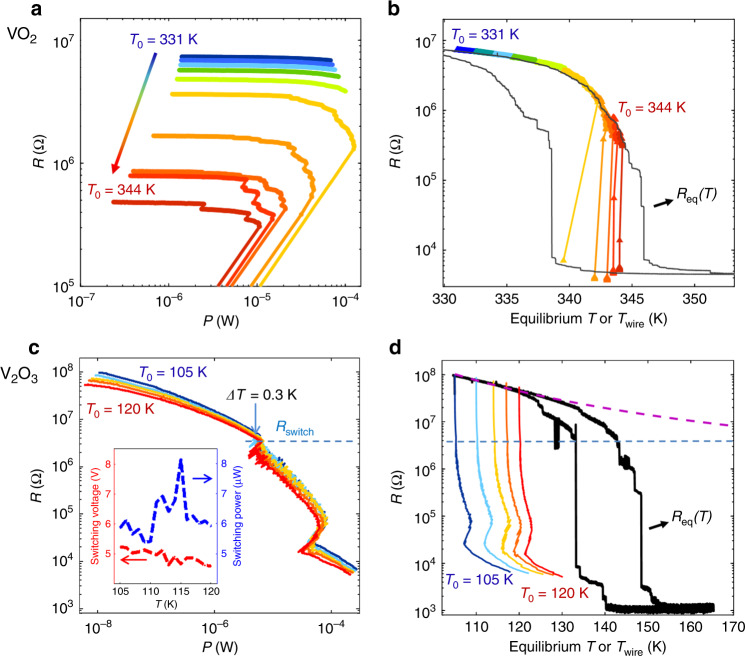
Thermal vs. non-thermal IMT switching. **a** and **c** *R*(*P*) curves acquired at different substrate temperatures, *T*_0_, corresponding to the insulating state of VO_2_ and V_2_O_3_ nanowires, respectively. The IMT switching is manifested as abrupt resistance jumps. **b** and **d** *R*(*T*_wire_) curves derived from the corresponding *R*(*P*) curves and superimposed on the *R*_eq_(*T*) (black). For the VO_2_ nanowire in **b**, the excellent correspondence between *R*(*T*_wire_) and *R*_eq_(*T*) signifies that the IMT is triggered thermally. For the V_2_O_3_ nanowire in **d**, no such correspondence between *R*(*T*_wire_) and *R*_eq_(*T*) is observed, showing the inadequacy of a Joule-heating driven mechanism. The IMT switching in **c** begins with an increase of temperature Δ*T* = 0.3 K in *T*_wire_ regardless of *T*_0_ (light blue arrow), indicating a negligible role of Joule heating. The switching resistance *R*_switch_ (dashed blue line) is lower than the fully insulating state resistance at any temperature. This is shown in **d** by the extrapolation of *R*_eq_(*T*) according to thermally activated resistance behavior with an activation energy of 60 meV (purple dashed line). The inset in **c** shows the switching voltage and power as functions of *T*_0_.

To explore the thermal and electric field effects on the electrically induced IMT, current-controlled *V*(*I*) measurements were performed in the low-temperature insulating state of the nanowires. For each *V*(*I*), the sample was initially cooled below the IMT hysteresis regime and then heated to *T*_0_ to allow comparison with the heating branch of the *R*_eq_(*T*) measurement. The *R*(*P*) curves for the VO_2_ nanowire in the low-temperature insulating regime are shown in Fig. 2a. A series of abrupt resistance jumps is observed in the *R*(*P*) curves for *T*_0_ > 337 K (light green curve) and are due to portions of the nanowire undergoing the IMT due to the applied current. These switching events occur with decreasing power as *T*_0_ increases. Due to the nanowire geometry, filamentary conduction does not occur so that domains evolve similarly to a temperature-driven IMT (see Supplementary Note 1). This enables a comparison between the *R*(*P*) and *R*(*T*_eq_). Using only the *κ* value measured above the IMT, we used Eq. (2) to transform the *R*(*P*) curves measured at different *T*_0_ in the insulating state to *R*(*T*_wire_). Figure 2b shows that the *R*(*T*_wire_) curves collapse onto the *R*(T_eq_), similar to the collapse observed in the metallic state (see Fig. 1a, c). The collapse, extending well into the IMT, shows that electrical switching in this VO_2_ nanowire can be accounted for solely by Joule heating. These findings are consistent with most previous reports which suggest that Joule heating plays a dominant role in the electrically induced IMT in VO_2_^21,30,39,40^. The large jumps in the last section of some *R*(*T*_wire_) curves are attributable to thermal runaway effects due to a current surge in the nanowire (see Supplementary Note 3 and footnote 1).

We note that our temperature calibration is strictly valid only in the homogeneous states (fully metallic or insulating). However, it is found that *R*(*T*_wire_) collapses onto the *R*_eq_(*T*) even when some metallic domains are present in the mostly insulating nanowire. This indicates that the temperature is approximately constant along the nanowire despite spatial variations in heat dissipation. This is due to thermal diffusion within the nanowire which tends to smear temperature variations. For higher power/temperature most of the sample is metallic resulting in a highly inhomogeneous temperature profile. To adequately describe this state local dissipation and spatial derivative terms have to be considered. However, for the purpose of identifying the mechanism behind the electrical IMT, we focus on the initial jump in the low power/temperature regime where Eq. (1) is valid.

Considering the electrically induced IMT switching shown in Fig. 2a, b as a benchmark for a purely Joule heating driven transition, we show that the V_2_O_3_ sample discussed in Fig. 2c, d exhibits pronounced qualitative differences. To assess the role of Joule heating, we follow the same procedure as before to calculate *R*(*T*_wire_) from the measured *R*(*P*) in the metallic state and Eq. (2). The *R*(*P*) and corresponding *R*(*T*_wire_) curves in the insulating state of this V_2_O_3_ nanowire are shown in Fig. 2c, d, respectively. The stark differences between these results and those of the VO_2_ sample shown in Fig. 2a, b, indicate that the thermal model is inadequate for describing the evolution of resistance with power in this V_2_O_3_ nanowire. In the following, we show that this is due to two phenomena which occur with negligible change in temperature: a smooth decrease in resistance and subsequent abrupt resistive switching. Initially, *R*(*P*) decreases smoothly by over an order of magnitude with increasing power up to ~5–8 μW. As can be deduced from comparison of the *R*(*T*_wire_) to *R*_eq_(*T*), the voltage-driven decrease in resistance is not due to Joule heating, since the dissipated power is too low (Fig. 2d). This smooth decrease is not attributed to the IMT either, but rather to field-assisted carrier generation, as will be discussed subsequently. After *R* decreases smoothly down to 3–5 MΩ it drops abruptly by 20–30%. It is tempting to immediately attribute this abrupt resistance drop to the IMT, but before we may safely do so, we have to rule out other possibilities. Besides an electrically driven IMT, resistive switching can be due to ionic migration, thermally induced negative differential resistance (NDR), and ovonic switching. Our recent work demonstrated oxygen migration in vanadium oxides under high applied electric field^10^. This process changes the material stoichiometry resulting in abrupt non-volatile resistive switching, which can only be reversed by high-temperature annealing. In contrast, the resistive switching in our device is volatile at temperatures below the IMT hysteresis (i.e. the initial resistance recovers when the current is ramped down), thus ruling out oxygen migration as the cause for the resistance jumps. Thermally induced NDR can also result in abrupt resistive switching before the onset of IMT in materials such as NbO_2_^41,42^. Such an NDR is due to a steep resistance-temperature dependence and appears with very significant Joule heating (Δ*T* ~ 100 K). In our devices, we deduce that the temperature increases by only ~0.3 K from the power-to-temperature calibration (see also details on Joule heating dynamics in our samples in Supplementary Note 3) (see footnote 1), which is inconsistent with a thermally induced NDR. Finally, non-thermal ovonic switching can also produce an abrupt resistance jump^43^. This type of threshold switching is due to carrier generation and saturation of charge traps under the application of a strong electric current. When the current is ramped down below a certain holding current, the device reverts back to the high resistance state. However, when switching within the hysteretic temperature range of our devices, the initial high resistance state is not recovered, even at zero applied current. This shows that portions of the nanowire have undergone the IMT and remain in the metallic state because it is thermodynamically stable at these temperatures. After ruling out other possibilities for the observed threshold resistive switching, we conclude that the resistance jumps in Fig. 2c are indeed due to an electrically driven IMT.

As noted above, the power just before the first switching event (~7 μW) heats the nanowire by only ~0.3 K, despite a 15 K range in *T*_0_. Moreover, the switching power is nearly independent of *T*_0_, and varies in the range 5–8 μW (see inset in Fig. 2c). These findings are inconsistent with Joule heating-driven switching, for which the switching power would decrease with increasing *T*_0_ by ~21 μW K^−1^ (as deduced from power-to-temperature calibration). We therefore conclude that the switching observed in this sample is due to a non-thermal, field-driven IMT.

**Fig. 3:**
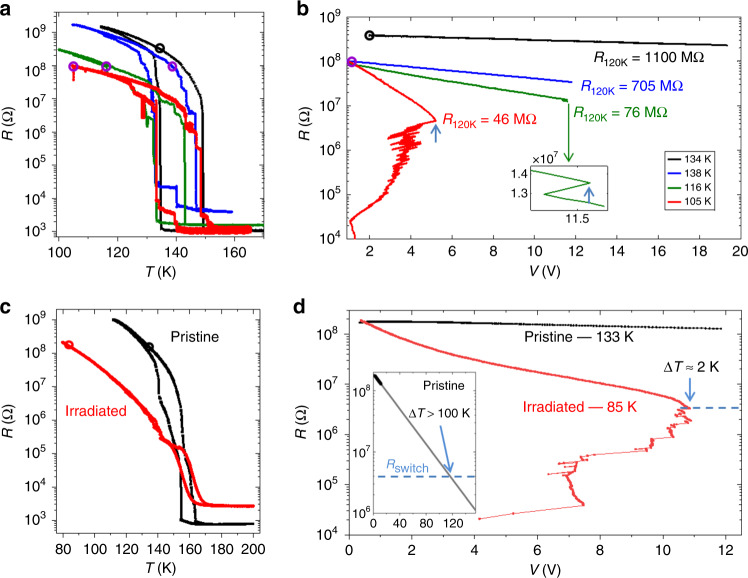
Influence of defects on the switching mechanism. **a** Resistance vs. temperature curves of four V_2_O_3_ nanowires. Open circles denote *T*_0_ for which the *R*(*V*) curves shown in **b** were acquired. **b** *R*(*V*) for each of the nanowires in **a** with the corresponding color coding. *T*_0_ is indicated in the legend. Samples with lower insulating state resistance, *R*_120K_, exhibit steeper slopes of log(*R*) vs. voltage, indicating enhanced carrier generation. For two samples, abrupt resistance jumps associated with the IMT are observed (light blue arrows). **c** *R*_eq_(*T*) before and after focused ion beam irradiation with a dose of 6.2 × 10^14^ Ga ions cm^−2^. Open circles denote *T*_0_ for which the *R*(*V*) curves shown in **d** were acquired. **d** *R*(*V*) measured in the pristine and irradiated states. Similar to **b**, the slope of log(*R*) vs. voltage is considerably steeper in the irradiated state. After the smooth decrease, the resistance reaches 2% of the initial value, *R*_switch_ = 4 MΩ, followed by non-thermal switching with a temperature increase Δ*T* ~ 2 K. For the pristine state, assuming the same *R*_switch_ = 4 MΩ, an extrapolation of *R*(*V*) predicts that switching occurs with over 110 V corresponding to a temperature increase of more than 100 K (inset to **d**).

Interestingly, the resistive switching properties of different V_2_O_3_ nanowires showed some variability, most likely induced during the fabrication process. A systematic comparison of these properties allowed us to gain insight into the underlying mechanism of the electric-field-driven IMT. Figure 3a shows *R*_eq_(*T*) curves of four V_2_O_3_ nanowires. The IMT in all four nanowires occurs over virtually the same temperature range. We find, however, that for any fixed temperature below the IMT hysteresis, the equilibrium insulating-state resistance varies between the nanowires. For instance, the resistance at 120 K (*R*_120K_) in the heating branch of the *R*_eq_(*T*) curves varies from 46 to 1100 MΩ. Since the nanowire geometries are nearly identical, these resistance variations are most likely due to properties of defects. Defects may create in-gap trap states that release carriers by thermal activation. Thus, the defect concentration and activation energy determine the amount of carriers which can be excited into the conduction band, thereby strongly influencing the insulating state resistance^44^. Indeed, recent studies have shown that defects due to deviations from perfect stoichiometry decrease the insulating state resistivity in VO_2_ and V_2_O_3_^45,46^. In our V_2_O_3_ samples we also find that *R*_120__K_ increases monotonously with the activation energy (see Supplementary Note 4 and footnote 1). We thus use *R*_120K_ as an inverse proxy for the amount of carriers which can be generated in the nanowires.

Based on this analysis, we now show that defects play a crucial role in determining the non-equilibrium properties of V_2_O_3_ and, consequently, affect the mechanism by which the IMT takes place. To elucidate this, typical *R*(*V*) curves for the samples of Fig. 3a are shown in Fig. 3b. For comparison, three *R*(*V*) curves were measured at different *T*_0_ so that they have the same initial resistance (blue, green, and red). This eliminates the influence of initial resistance and enables a fair comparison between the samples. Two important effects are observed for samples with lower *R*_120K_: (1) the initial slope of the smooth part of log[*R*(*V*)] curve becomes steeper and (2) the switching voltage (*V*_switch_) decreases. Unexpectedly, we find that *V*_switch_ is lower in measurements performed at lower temperatures, as shown in Fig. 3b. This points to the dominant role of defects in determining switching properties rather than the proximity of *T*_0_ to *T*_IMT_. For instance, the sample with *R*_120K_ = 46 MΩ switches with only ~5.2 V (1.5 MV m^−1^) and ~6 μW at *T*_0_ = 105 K, well below *T*_IMT_. In contrast, the sample with *R*_120K_ = 705 MΩ does not switch up to 12 V (3.4 MV^−1^) with similar applied power at *T*_0_ = 138 K, despite being inside the phase coexistence regime. The sample with the highest *R*_120K_ = 1100 MΩ does not switch even with application of 20 V. The non-equilibrium properties are thus observed to be strongly dependent on the insulating state resistance, which is strongly affected by defects in the nanowire.

To examine this further, we used focused Ga ion beam irradiation to systematically and controllably create defects in our samples. Only the nanowire area was exposed to the ion beam with minimal exposure to the contacts, so that the resulting changes in transport properties can only be attributed to the nanowire itself. Figure 3c shows the *R*_eq_(*T*) curves of a V_2_O_3_ nanowire before and after ion irradiation. Similar to the samples shown in Fig. 3a, defects reduce the insulating state resistance but do not considerably affect the temperature range over which the IMT takes place. As in the non-irradiated samples, the *R*(*V*) characteristics show dramatic differences due to the varying defect density. Figure 3d shows two *R*(*V*) measurements acquired at *T*_0_ = 133 and 85 K in the pristine and irradiated states, respectively, so that the initial resistance is the same in both cases. For the pristine state, the resistance smoothly decreases to 73% of the initial value for the maximum voltage, while the resistance of the irradiated sample smoothly decreases by almost two order of magnitude to ~2% of its initial value, followed by an abrupt resistance jump at *R*_switch_ = 4 MΩ (light blue arrow). Just before switching, Joule heating in the nanowire corresponds to a temperature increase of <2 K (*T*_wire_ < 87 K). However, the first signatures of the IMT in *R*_eq_(*T*) occur at ~120 K, which would require an increase in *T*_wire_ of ~35 K. Therefore, this switching cannot be explained by thermal effects. Qualitatively similar results were obtained also on irradiated VO_2_ nanowires (see Supplementary Note 5 and footnote 1), thus corroborating the important role of defects in facilitating the electrically driven non-thermal IMT in Mott insulators.

## Discussion

Zener-tunneling is a well-studied mechanism for field-induced doping and destabilization of Mott insulators^47–51^. Within this mechanism, the electric field is high enough (70–400 MV m^−1^) to enable tunneling of carriers across the Mott gap, which drives a doping driven transition. In our work, the switching fields are much smaller (<3 MV m^−1^) so that Zener-tunneling can be ruled out. However, a similar end result can be achieved by field-assisted activation of carriers from trap-states. Instead of tunneling through the gap, charge trapped in defect in-gap states can be activated and provide enough carriers to dope the Mott insulator and induce the IMT. Previous theoretical works have shown that impurities can dramatically reduce the switching field with respect to the one expected from Zener tunneling^52,53^. In the following, we discuss how defects increase field-assisted carrier emission in our samples, which in turn enables a non-thermal doping-driven IMT with low field.

**Fig. 4:**
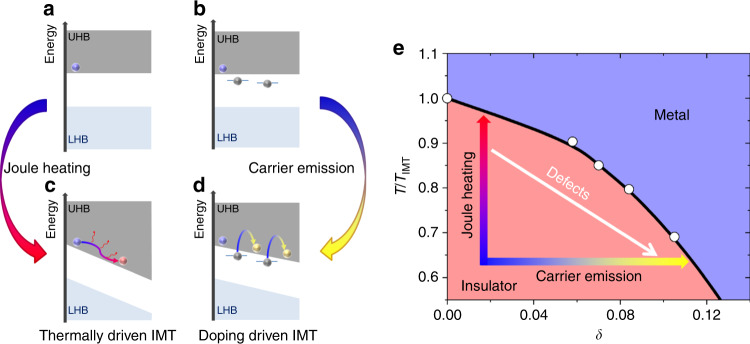
Two routes for resistive switching in a Mott insulator. **a**–**d** Schematic representation of Joule heating and field-driven IMT switching in a Mott insulator. In a system with no defects **a**, only a small number of thermally activated carriers are present in the upper Hubbard band (UHB). Application of a strong electric field, depicted as strong band tilt **c**, accelerates these carriers causing Joule heating due to scattering. In a system with defects **b**, a moderate electric field promotes carriers from in-gap states into the UHB **d** resulting in collapse of the Mott insulator state via a doping-driven IMT. **e** Phase diagram of the single band Hubbard model based on DMFT calculations. The colored arrows schematically denote the two possible routes towards the metallic phase when applying electric field as discussed in **a**–**d**. Defects increase the effectiveness of carrier emission and facilitate a doping driven non-thermal IMT.

First, we discuss the origin of the initial smooth decrease in resistance in response to the applied voltage as shown in Fig. 3b, d. This decrease cannot be attributed to the IMT since switching even in a single domain would produce a resistance jump due to the low dimensionality of the nanowire, which is not observed. Furthermore, hysteretic behavior is not observed in the *R*(*V*) curves within the initial voltage-driven smooth decrease of *R*, even at temperatures within the phase coexistence regime. Similar voltage-driven decrease in resistance has been previously observed in Mott insulators^20,54^ and attributed to field-assisted carrier generation. One mechanism for this is the Poole–Frenkel effect^55^, in which the electric field reduces the energy barrier for excitation of trapped carriers from in-gap states into the upper Hubbard band (Fig. 4b, d). We note that only carrier excitation into the upper Hubbard band is shown in Fig. 4 but carriers may also be excited into other unoccupied bands. For example, in low-temperature insulating V_2_O_3_, the a_1g_ band, which belongs to the lower Hubbard bands, is above the Fermi level and, consequently, is unoccupied^56^. As a function of the electric field (*E*), the energy barrier may decrease linearly with *E* or *E*^1/2^ ^57^, producing a large increase in carrier density, consistent with the observed decrease of resistance with voltage (see also Supplementary Note 6 for details and fitting to the Poole–Frenkel conduction enhancement) (see footnote 1). The slope of these curves steepens with decreasing *R*_120K_, indicating that defects determine the number of carriers which are excited for the same applied field^58^. As demonstrated in Supplementary Fig. 6 (see footnote 1), the resistance just before switching (*R*_switch_) is nearly independent of *T* and lower than the equilibrium resistance of the fully insulating phase, even if it is extrapolated to temperatures higher than that of the IMT (Fig. 2d). Therefore, at *R*_switch_, the effect of field is to increase the carrier concentration beyond that of the insulating state in equilibrium. This excess carrier concentration leads to destabilization of the insulating state, resulting in emergence of the metallic state. Since field-assisted carrier concentration increases with temperature, the switching voltage (*V*_switch_) decreases with increasing *T*_0_ (inset to Fig. 2c). We note that other field-enhanced carrier emission processes may also lead to similar results. For instance, switching in the Ga-ion-irradiated VO_2_ nanowire is preceded by a conductivity enhancement according to ${\mathrm{ln}}({\sigma}) \propto {E}^2$, indicative of phonon-assisted tunneling^59^ (see Supplementary Note 6 and footnote 1).

In samples where the electric-field-assisted carrier generation is inefficient, the doping driven transition cannot be attained even for the highest applied voltages and the only possible route to induce the IMT switching is through Joule heating past *T*_IMT_ (Fig. 4a, c). This is most notable for the non-irradiated VO_2_ nanowires which transition thermally (Fig. 2a, Supplementary Fig. 4 and footnote 1) and non-irradiated V_2_O_3_ nanowires with the highest insulating state resistances (black curves in Fig. 3b and d). For these V_2_O_3_ samples, even up to the highest applied voltages, the resistance decreases very little compared to samples with low *R*_120K_. Therefore, the carrier density does not reach the critical value required to induce switching in a non-destructive voltage range. We therefore assess *V*_switch_ in these samples by extrapolation. For instance, the inset to Fig. 3d shows a linear extrapolation of the log[*R*(*V*)] for the pristine nanowire to a resistance of 4 MΩ—the *R*_switch_ value observed in the same sample after irradiation. Assuming that the linear dependence holds for higher voltages, switching would occur with over 110 V. This translates to Joule heating by over 100 K, which would bring the sample well above *T*_IMT_. This indicates that thermal effects would play a major role in the electrical triggering of the IMT in this sample. We therefore conclude that there is a crossover in the switching mechanism from carrier generation to Joule heating in both VO_2_ and V_2_O_3_.

We note that non-thermal switching in VO_2_ requires higher irradiation doses compared to V_2_O_3_ (see Supplementary Note 5 and footnote 1). Even with these high doses, electric-field-assisted carrier generation was not as effective as in V_2_O_3_, leading to higher *V*_switch_ values in VO_2_. This points to the role of the activation energy which is considerably higher in VO_2_ (265 meV) compared to the V_2_O_3_ samples which exhibit non-thermal switching (60 meV). *V*_switch_ also increases with increasing activation energies for the various V_2_O_3_ samples shown in Fig. 3 (see Supplementary Note 4 and footnote 1). We thus conclude that with higher defect densities and lower activation energy, more carriers are generated with less applied field and power, which enables non-thermal switching. In the clean limit, carrier generation is inefficient and Joule heating thermally triggers the IMT before the critical carrier concentration is attained (Fig. 4).

The strong dependence on specific sample properties can explain apparent discrepancies regarding the nature of the IMT found in previous reports on the same materials^20,21,25,40,60^. These reports may be reconciled by considering differences in sample properties such as resistivity, permittivity, defect density, and activation energy (see also Supplementary Notes 2, 6 and footnote 1). These properties are crucial for determining whether a Mott insulator will undergo thermal or non-thermal electrical switching. We note that the quasi-1D nature of our samples helps to identify the mechanism behind the electrically driven IMT by eliminating filament formation (see also discussion in Supplementary Note 3 and footnote 1). However, the sample geometry does not determine the switching mechanism. Geometry might affect the overall switching dynamics and total energy consumption but not its microscopic origin. In fact, several studies have concluded based on simulations and switching time scales that in 2D/3D devices the transition temperature is not attained during switching^21,25,27^.

To gain deeper insight into the physics of the electrically induced IMT, we performed dynamical mean field theory (DMFT)^61^ calculations for a single band Hubbard model. This model is considered the simplest framework for a qualitative description of the IMT in a strongly interacting electron system (see Supplementary Note 6 and footnote 1). Motivated by our previous discussion, we assume that the main effect of the electric field is to promote charge carriers in the system, which is modeled by shifting the electric (chemical) potential to adjust the doping level. Starting from the insulator state, the resistivity *ρ*(*T*) was calculated using the Kubo formula for various temperatures and doping levels to determine the regimes of insulating (d*ρ*/d*T* < 0) and metallic (d*ρ*/d*T* > 0) behavior. The goal is to qualitatively examine the resistivity across the thermal and doping-driven IMT and compare these results with our experimental findings. The calculations show that the IMT can be attained either by increasing temperature or by doping (see phase diagram in Fig. 4e). This is qualitatively consistent with the phase diagram of many Mott insulators including the cuprates^62^, VO_2_^63^, and V_2_O_3_^64^_,_ even though these materials have a more complicated band structure than that of our model. Despite the simplicity of the model, several important characteristics of the experimental switching are reproduced by our calculations. We observe that *ρ* decreases considerably as the sample is doped by a few percent until the system switches from insulating to metallic behavior. Moreover, it is found that *ρ* at the switching point is lower than that of the insulating state in equilibrium and has a weak dependence on temperature, as we observe experimentally (see Supplementary Note 6). The ability of such a general and schematic model to capture important aspects of our experimental findings suggests that the proposed mechanism for the electric field-driven IMT may apply to many Mott insulators beyond VO_2_ and V_2_O_3_.

The ability to induce non-thermal switching without heating the device by more than a fraction of a Kelvin implies that this process can be highly energy efficient. Indeed, by applying nanosecond voltage pulses to the V_2_O_3_ nanowire discussed in Fig. 2c, d, an upper bound of 5 fJ is found for the switching energy (Supplementary Note 7). This presents an improvement in energy consumption of about three orders of magnitude over the Joule heating-driven IMT and rivals resistive switching in state of the art memristors^65^. This has very important implications on the energetics of devices, which utilize the IMT for memory or for emulating neural functionalities. Moreover, the time scales associated with the non-thermal IMT may be much shorter than for the thermal IMT. For devices based on a thermal IMT, accumulation and dissipation of heat sets a lower bound on the operation time scales^66^. By avoiding heating during the switching process these time scales may be greatly reduced.

To conclude, in this work we disentangle the effect of Joule heating and electric field on resistive switching in two archetypal Mott insulators—VO_2_ and V_2_O_3_. We show clear evidence for thermal and non-thermal IMTs in both materials. Non-thermal switching occurs when field-assisted carrier generation leads to critical doping levels where the Mott insulator state is no longer stable. Several features of our experimental findings are reproduced by DMFT calculations, supporting the doping-driven IMT scenario. We find that defects are crucial for the non-thermal IMT, as they provide the charge reservoir for the doping process. To enhance their effect, large defect densities may be introduced into the sample during fabrication or by focused ion beam (FIB) irradiation. This allows moderate fields to excite a large number of carriers with negligible heating. For low defect densities, carrier generation is unable to attain critical values, leaving Joule heating as the dominant switching mechanism. Our work demonstrates that, despite having two different ground states, VO_2_ and V_2_O_3_ can be modified through defect engineering to exhibit a non-thermal IMT. This suggests that using controlled defects to induce non-thermal switching may be applicable to many Mott insulators, and greatly reduce the energy consumption and operating timescales of IMT-based devices.

## Methods

### Sample preparation

*VO*_*2*_: A 100 nm-thick VO_2_ film was grown on (012)-oriented Al_2_O_3_ substrate by reactive rf magnetron sputtering. The growth was done in a 4 mTorr Ar/O_2_ (92–8%) atmosphere and at a substrate temperature of 470 °C. After the growth, the sample was cooled down at a rate of 12 °C min^−1^. X-ray diffraction showed textured growth along the (100) direction of VO_2_. *V*_*2*_*O*_*3*_: 300 nm-thick V_2_O_3_ films were grown on (012)-oriented Al_2_O_3_ substrates by rf magnetron sputtering. The growth was done in a 8 mTorr Ar atmosphere and at a substrate temperature of 700 °C. After growth, the samples were thermally quenched at a rate of ~90 °C min^−1^, which significantly improved the film’s electrical properties^67^. X-ray diffraction showed textured growth along the (012) direction of V_2_O_3_.

For both materials, nanowires of 85–100 nm width were fabricated by e-beam lithography and reactive ion etching. Au (100 nm)/Ti (20 nm) electrodes were subsequently prepared by photolithography and e-beam evaporation to electrically contact 3–3.5 μm long nanowires.

### Transport measurements

Transport measurements were performed in a probe station using a current source pulse generator, nanovoltmeter, and 20 GHz oscilloscope. To protect the sample against current surges during abrupt resistive switching, for some measurements a resistor was connected in series. Fast transport measurements to determine the energy consumption during non-thermal switching were performed using a bias tee to mix nanosecond pulses with a small DC current. The DC current was used to probe the resistance of the sample before and after the pulse. Due to the large impedance mismatch, a 50 Ω termination was added at the output of the pulse generator to avoid reflection.

### FIB irradiation

Ion beam irradiation was performed in a FEI Scios DualBeam system using a FIB of 30 keV Ga ions. The etch rate was calibrated by measuring the dose required to etch the vanadium oxide film all the way to the substrate. For the dose used in this work (6.2 × 10^14^ ions cm^−2^), the etching depth is estimated at ~1.4 Å, so that the film thickness is practically unaffected. The nanowire region was irradiated by scanning the FIB at 1.5 pA with 50% overlap between successive locations to ensure a uniform irradiation dose across the entire nanowire.

## Supplementary information

Supplementary Information

## Data Availability

All relevant data is available from the corresponding author upon request.

## Footnotes

1 See Supplemental material at [<#] for details of sample preparation, measurement procedures, simulations, DMFT calculations and supporting experiments.

## Code availability

All code used in simulations supporting this article is available from Y.K. (ykalchheim@ucsd.edu), J.d.V. (jdelvallegranda@ucsd.edu), or M.R. (marcelo-rozenberg@u-psud.fr) upon request.
