# Supplementary material for: Non-thermal resistive switching in Mott insulator nanowires
Source: Nat Commun. 2020 Jun 12;11:2985. doi: 10.1038/s41467-020-16752-1 (PMC7293290; doi:10.1038/s41467-020-16752-1)
Supplement: Supplementary file 1 — Supplementary Information [file 41467_2020_16752_MOESM1_ESM.pdf]

Supplementary Information

Non-Thermal Resistive Switching in Mott Insulator Nanowires

Kalcheim et al.

Supplementary Notes

Supplementary Note 1: *R* vs. *T* simulations and domain/filament size

To determine whether switching is thermally driven or not one must take into account the current and temperature distribution in the device, which may be highly spatially inhomogeneous. To understand the reason for this, the differences between the electrically-driven and temperature-driven IMT should be considered. When the sample temperature is raised uniformly across the IMT, a VO_x thin film will phase separate into metallic and insulating domains.[1] Metallic domains will nucleate and grow at the expense of insulating ones until the sample is fully metallic. If the typical size of such domains is small compared to the size of the device, a percolative transition will take place. In contrast, when driving the device with current, certain paths connecting the electrodes will have a lower resistance and they will attract more current and power than others. Eventually, a continuous path of metallic domains will form between the electrodes, manifesting as a sharp decrease in resistance and virtually the entire current will flow through a filament, while the rest of the sample will remain in the insulating state. With increasing power the filament will grow until the entire device is in the metallic state. During this process, the temperature in the device may be highly inhomogeneous and hard to measure or calculate. This makes it hard to determine if a transition can be induced without reaching the IMT temperature. Moreover, once a filament forms, the electric field on the device drops abruptly and only thermal effects from the heat injected through the filament will be observed subsequently. Therefore, when studying the importance of thermal effects on the IMT it is desirable to avoid filamentary conduction. This can be achieved by studying nanowires in which the domain size is comparable to the width of the nanowire.

For V_2O_3, thicknesses of 300 nm produce large domains, several hundreds of nanometers wide.[2] This large domain size is a result of high domain interface energy compared with thinner films.[3] The thickness of the VOx was selected so that the domain size is comparable or larger than the nanowire width. This manifests as large jumps in the $R_{eq}(T)$ curve, as shown in Supplementary Fig. 1(a,b). When the size of the domains approaches the nanowire width the jumps become larger. From comparison with simulations of our $R_{eq}(T)$ curves we find that typically, 1-2 domains are present in a lateral cut of the nanowire (see [4] for simulation details). Thus, the inhomogeneous current and temperature distributions which may occur in 2D devices are avoided.

Given the quasi-1D geometry, filamentary conduction during the V(I) measurements is highly unlikely in our samples as we discuss in the following. For both VO_2 and V_2O_3, after the first switching event, the resistance is around ~1000 times that of the purely metallic phase. If this resistance corresponds to filamentary conduction, the cross section of the filament would be ~3-4 nm. This is much smaller than the typical size of domains in our sample as deduced from the $R_{eq}(T)$ simulations. However, filaments in VO_2 and V_2O_3 devices of similar thickness were found to be ~1 μm wide [5,6] which exceeds the typical domain size. We can thus rule out the formation of a conducting path between the electrodes, at least in the first abrupt resistance drop. Moreover, since the evolution of domains with temperature results in a very different domain configuration than that of filamentary conduction, one does not expect the observed correspondence between the $R(T_{wire})$ and $R_{eq}(T)$ shown in Fig. 2(b) of the main text.

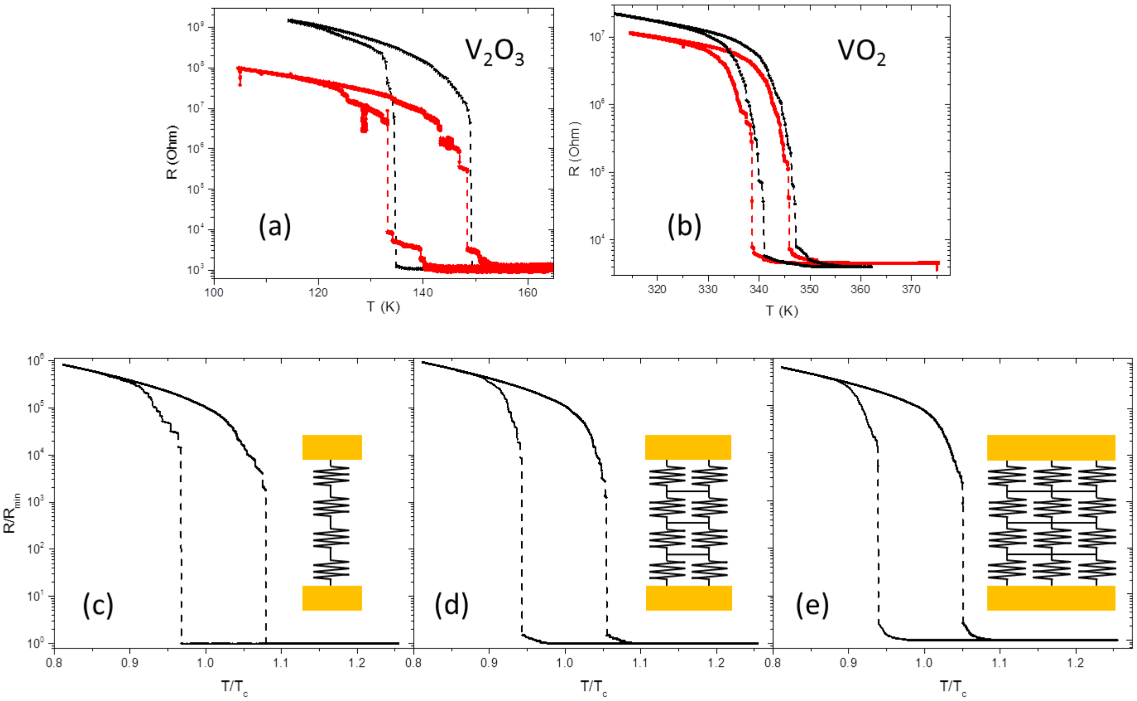

Supplementary Fig. 1: (a) and (b) Measured resistance vs temperature curves for V_2O_3 and VO_2 nanowires. (c), (d) and (e) - Simulated R(T) curves for 1x20, 2x40, 3x60 resistor networks respectively. The biggest difference between the simulations is observed in the low resistance regime where the resistance jumps have the most impact on the shape of the curve. A one dimensional resistor chain has one abrupt jump to high resistances from the fully metallic state as observed experimentally in the black curve in (a) and in the simulation (c). The curves become smoother when the resistor network has more resistors per lateral cut.

Supplementary Note 2: Comparison to previous work

We have noticed that, in contrast to nanowires, continuous films such as the ones used in [7] show significantly less field assisted carrier generation, resulting in a dominantly Joule heating driven transition. This is probably due to the nanowire

etching process which creates defects in the nanowire, similarly to the Ga irradiation treatment. Since most studies are conducted on continuous films, defect densities may be too low to observe non-thermal switching.

Another possible issue is that the thermal coupling constants found in the present study are almost an order of magnitude larger than those estimated in [7]. There, κ was deduced from the minimal power required to electrically maintain a highly conducting state below T_c in a V_2O_3 junction. However, in this highly conducting state the current did not flow through the entire V_2O_3 sample. Therefore, this method produces a lower bound on κ, since maintaining the entire junction metallic requires significantly more power. This may explain why the conclusions of that study differ from the ones presented here, and highlights the importance of using 1D nanowires to accurately measure thermal coupling constants.

Supplementary Note 3: Joule heating dynamics

We note that previous work on V_2O_3 devices suggests that a thermal runaway effect may inhibit the determination of T_{wire} prior to a switching event in a dc measurement.[7] This effect is due to the decrease in resistance with temperature *in the insulating state before any metallic domains form*. Thus, for constant voltage, this may result in a positive feedback loop whereby increasing power decreases the resistance, which increases the power further. This effect has recently been observed also in NbO_2 well below its Mott transition temperature.[8] Due to parasitic capacitance, during switching, a transient current surge may occur in our devices which could result in a similar thermal runaway effect. To check if such an effect can apply to our measurements, we performed simulations using the experimentally measured parameters in the heat equation assuming that the voltage

is constant even when the resistance goes down. This yields the following equation:

$$C\frac{dT_{\mathrm{wire}}}{dt} = \frac{V^2}{R(V,T_{\mathrm{wire}})} + \kappa(T_0 - T_{\mathrm{wire}}) \qquad (1)$$

$R(V,T_{\mathrm{wire}})$ is determined from *R(V)* measurements acquired at different temperatures with an activated behavior of the form (see section 4)

$$R(V,T_{\mathrm{wire}}) = R_0(V)\exp(\varDelta/k_B T_{\mathrm{wire}}) \qquad (2)$$

In our simulations we used the heat capacitance $C=1.46\cdot10^{-13}$ J K^{-1} derived from the literature value of (~35 J mol^{-1} K^{-1}).[9] However, since the heat capacitance can be absorbed into the time step, the stability analysis does not depend on its value, and all other parameters are measured. Our simulations show no thermal runaway effects when using the measured parameters, V=5.3 V, κ=21 μW/K and $\varDelta$=60 meV. To reach instability by varying one of the parameters, the above values had to be changed to V=23 V (6.5 MV/m), κ=0.7 μW/K and $\varDelta$=2000 meV respectively (see Supplementary Fig. 2). Thus, thermal runaway effects in the insulating state can be excluded in our samples and measurement conditions. This is further corroborated by the pulsed measurements discussed in the last section of the extended data. We note that in [7] a large contribution from Joule heating is observed most clearly at low T where the switching fields are significantly larger than 6.5 MV/m so that thermal runaway effects may play an important role in that case.

The nanowire geometry is advantageous for avoiding power surges and thermal runaway when a single metallic domain nucleates. If we consider switching of domains from the insulating to the metallic state, thermal runaway effects can be observed in our devices but only when resistance jumps represent a large fraction of the total resistance. In a nanowire, this occurs when only a few insulating domains are left so that the resistance would change substantially when one

domain switches. During thermal runaway, the sample temperature momentarily could exceed T_{IMT} prompting the insulator-metal transition. After thermal runaway is done, the sample would cool down from the fully metallic state, following the cooling brunch of the *R(T)*. Since these fast dynamics cannot be directly captured in dc IV measurements, only the final low resistance state would be registered. This explains the jumps in $R(T_{wire})$ down to the cooling curve values for the VO_2 nanowire (Fig. 2 in the main text). However, thermal runaway is not observed when resistance jumps represent only a small fraction of the total resistance of the nanowire. In such a case, the current surge is small and insufficient to trigger further switching of other domains. When a single domain switches, the remaining insulating domains in the nanowire act as a current limiter. This current limiting is made possible by the nanowire geometry, for which single domain switching changes the total resistance by a small fraction causing only a small current/field redistribution. This is rarely the case in the more common nano-gap geometry (see [7,10]). In the case of a nano-gap, nucleation of a metallic region reduces the sample resistance by a large fraction and causes a significant redistribution of current and field in the junction. This leads to switching of other domains so that the first resistance jump in such devices is often associated with filament formation and a power surge.

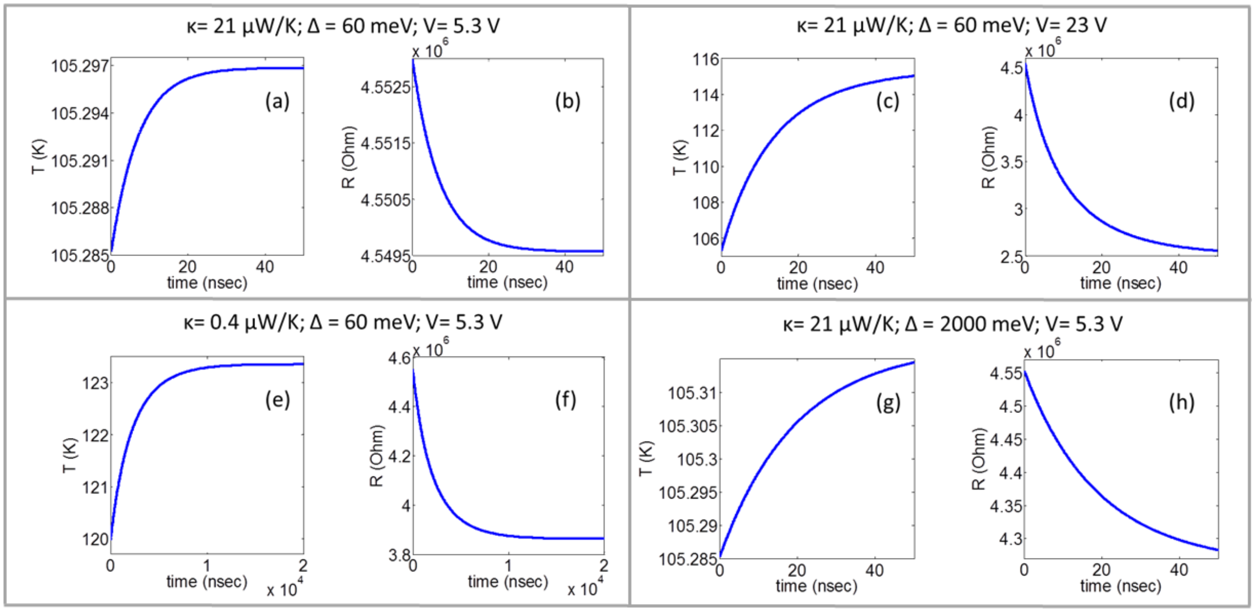

Supplementary Fig. 2: Simulated temporal evolution of the temperature and resistance following a voltage increase from 5.2 V to the specified value. This analysis shows the range of parameters for which no thermal runaway effects are attained in Supplementary Equation 1. The results for the measured parameters are shown in (a, b). These parameters are well within the stability range, indicating that thermal runaway effects within the insulating state are highly implausible in our samples.

Supplementary Note 4: Activation energies

Due to the strong dependence of the resistance on applied field for V_2O_3 the activation energies for the various samples were derived from resistance vs temperature curves acquired at a fixed voltage of 4 V (see Supplementary Fig. 3). The fit was performed to the low temperature part of the heating curve where no resistance jumps are observed so that the sample was completely in the insulating state at all fitted temperatures. As long as the sample is in the fully insulating state (no jumps observed in the *V(I)*), the activation energies are not sensitive to the choice of voltage. For VO_2 the fitting was performed to the *R(T)* curve acquired with constant current. For the 10^{-7} A applied during this *R(T)*, the deviation with respect to a constant voltage *R(T)* curve are negligible, as can be seen from the small change in resistance with voltages up to 14 V for the pristine sample shown in Supplementary Fig. 4.

Interestingly, we find that the activation energies vary considerably more for V_2O_3 nanowires than for VO_2, as do the switching characteristics. Lower activation energies correlate with enhanced carrier emission and lower switching voltage and power. Defect density and activation energy as well as other material properties seem to play a role in determining switching characteristics. See further discussion in sections 5 and 6.

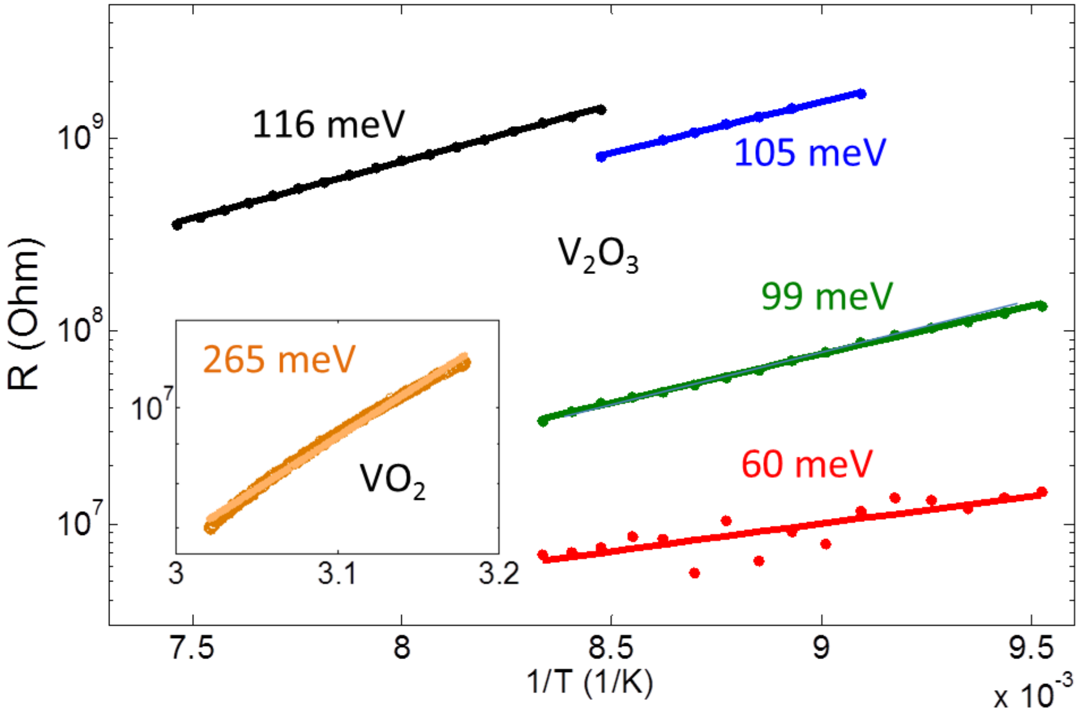

Supplementary Fig. 3: Activation energies for the V_2O_3 and VO_2 samples discussed in the main text according to $R(T) = R_0 \exp(\Delta/k_B T)$. The color coding of the V_2O_3 samples corresponds to Fig. 3(a) of the main text.

Supplementary Note 5: Irradiation effects in VO_2

A similar irradiation procedure to the one described in the main text was performed for VO_2 nanowires (see methods). Two *R(V)* curves are shown in Supplementary Fig. 4 for a nanowire irradiated with $6.2 \cdot 10^{15}$ ions cm^{-2} and a pristine nanowire. The R(V) for the irradiated sample is measured at 310 K, well below the IMT, whereas the R(V) for the pristine sample is measured at 335 K, within the phase coexistence regime (see arrows in Supplementary Fig. 4). The *R(V)* curve of the irradiated VO_2 nanowire shows a steeper slope compared to that of a pristine sample. As in V_2O_3, this shows that defects increase field assisted carrier generation. A subsequent

abrupt resistance drop occurs at ~13 V in the irradiated sample, signifying an electrically induced IMT. The power right before the switching is 23 μW which is equivalent to heating by ~0.5 K (see details of power calibration procedure in Fig. 1 of main text). However, the onset of the IMT is 15 K higher than T_0 (T_{onset}=325 K), thereby ruling out a thermally induced IMT. Despite being in the phase coexistence regime and applying similar voltage and power, the pristine sample does not show any switching.

Interestingly, when compared to V_2O_3, field assisted carrier generation is considerably less efficient in the case of VO_2 as can be deduced from the smaller change in resistance prior to switching for the same applied fields. This, in turn, results in higher electric fields required for switching. For instance, V_{switch} values of a few Volts were observed in V_2O_3 even in pristine samples, whereas all measured VO_2 samples showed V_{switch} values of over 12 V below the coexistence regime. This may be related to the differences in the magnitude of the activation energies, permitivitties and transition temperatures for both materials (see section 6 for further discussion). An open question remains, whether defect engineering may facilitate a higher degree of control over switching characteristics by tuning these material properties.

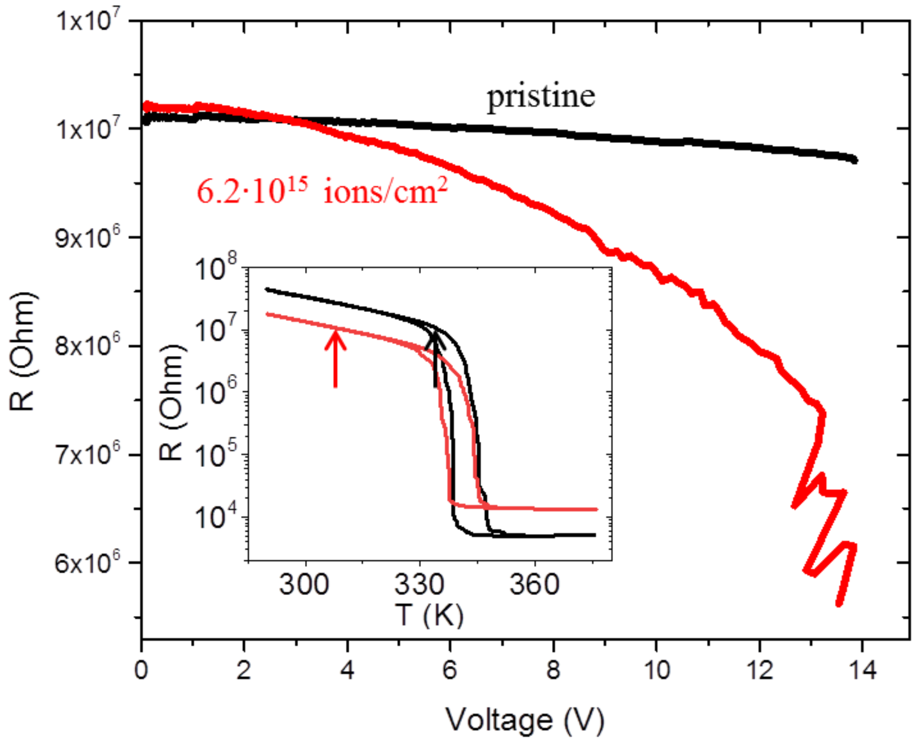

Supplementary Fig. 4: Resistance vs voltage measurement of a pristine VO_2 nanowire (black) and an ion irradiated VO_2 nanowire (red - $6.2 \cdot 10^{15}$ Ga ions/cm^2). The non-irradiated sample shows a small ~3% decrease in resistance at the highest V. The irradiated sample shows a considerably larger decrease in R with V and subsequent switching (abrupt resistance drops). The estimated increase in temperature at the first resistance drop is 0.5 K, whereas the IMT onset in equilibrium occurs more than 15 K above the measurement temperature. This shows the non-thermal nature of the resistive switching, as is the case in V_2O_3. The inset shows the R(T) for both nanowires along with the corresponding measurement temperatures.

Supplementary Note 6: Theoretical Modelling

Dynamical Mean Field Theory (DMFT)[11] provides a non-perturbative method to study the interplay between correlation effects and electron banding and has in particular produced insights into the Mott metal-insulator transition. DMFT theory maps a lattice problem onto a quantum impurity model, a finite-size system coupled to a non-interacting bath of electrons, plus a self-consistency condition. The simulations were performed using the continuous-time quantum Monte Carlo (CTQMC)[12], which samples a diagrammatic expansion of the partition function in powers of the impurity-bath hybridization. In this method the on-site Hamiltonian is solved exactly, and the coupling to the bath is treated by a perturbation expansion. We solve for the single band Hubbard model with an antiferromagnetic self-consistency condition,[11] which captures both the antiferromagnetic and paramagnetic solutions. For the non-interacting bath electrons we adopt a semi-circular density of states of half-bandwidth W = 1. Similar models have been employed for exploring the electrically driven IMT in previous theoretical studies.[13,14]

The calculations require solving the system across the thermal and carrier doping transition, in the very low doping regime. Furthermore, high precision is required for the calculation of the conductivity from Kubo formula. This is enabled by using CTQMC to obtain solutions of the Hubbard model at finite temperatures. We consider the model at intermediate correlation strength (U/W=1.7, where U is the Coulomb repulsion and W is the half-bandwidth), and examine the system at half-filling (i.e. one carrier per site) where it is, like V_2O_3, an antiferromagnetic insulator. Supplementary Fig. 5(a) displays the resistivity as a function of T where a second order IMT is observed, marked by a change in the sign of the slope of the $\rho(T)$ curve. In many Mott insulators, once the metallic state sets in, a structural

transition occurs simultaneously,[15] creating a large discontinuous decrease in resistance. For simplicity, the structural transition, along with the discontinuity in resistivity, is not included in this calculation since we are only interested in studying the destabilization of the insulating state with temperature/doping. We assume that the main effect of the electric field is to promote charge carriers in the system, which are described by doping the Mott insulator via an electric (chemical) potential.

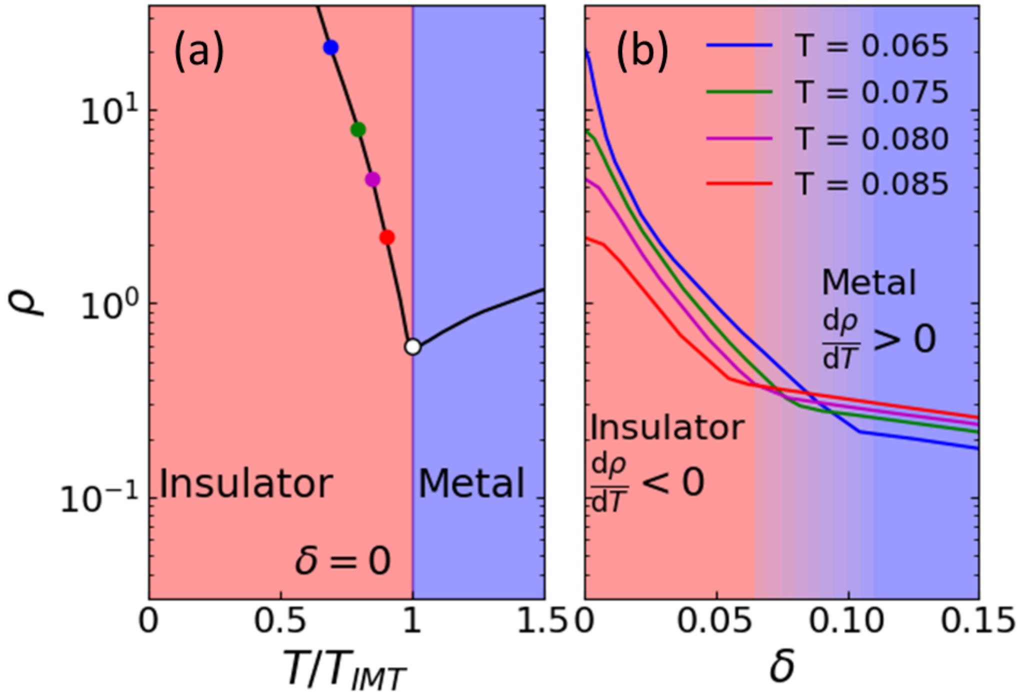

Supplementary Fig. 5: (a) resistivity as a function of temperature in the temperature driven insulator to metal transition at zero doping for a single band Hubbard model. (b) Doping driven transition for different starting temperatures showing a change from insulating (dρ/dT<0) to metallic behavior (dρ/dT>0). While ρ has a strong dependence on temperature at zero doping, the dependence of ρ at the doping driven IMT is only weakly T dependent, as observed experimentally.

Despite the simplicity of the model, several important experimental features are qualitatively reproduced by the calculation. Supplementary Fig. 5(b) shows the calculated resistivity as a function of doping for different T across the IMT. For low doping $d\rho/dT<0$ as expected in the insulating phase and ρ is strongly temperature dependent. As δ increases the temperature dependence weakens until the resistivity curves cross each other and at high doping $d\rho/dT>0$ (metallic state) for the entire modelled temperature range. Despite the strong dependence of $\rho(\delta=0)$ on T, the resistivity at the critical doping (ρ^*) is only weakly temperature dependent. This is consistent with the experimental observation that $R_{\mathrm{switch}}(T_0)$ is nearly independent of T_0, while larger temperature dependence is observed for $R_{\mathrm{eq}}(T)$ (see Supplementary Fig. 6).

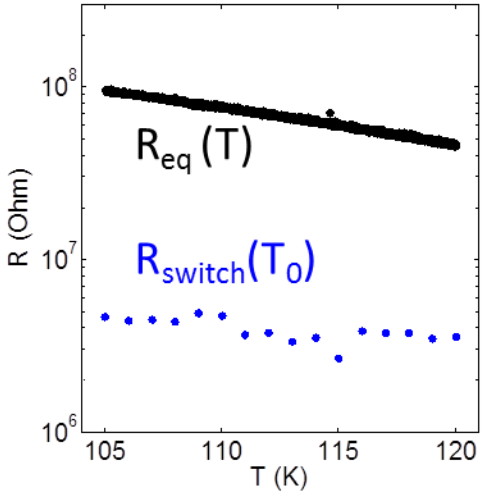

Supplementary Fig. 6: $R(T)_{eq}$ and switching resistance $R_{switch}(T_0)$ for the nanowire with R_{120K}=46 MΩ discussed in the main text. The horizontal axis refers both to the temperature during the equilibrium measurement and the different stage temperatures T_0 during V(I) sweeps.

The calculations also show that for all T, $\rho^*(\delta>0) < \rho^*(\delta=0)$. This is also observed experimentally as shown by the comparison between R_{switch} and the extrapolation of $R_{eq}(T)$ in the insulating state to temperatures above T_{IMT} (see Fig. 2(d) in the main text). This can be explained by considering that an increase in δ from zero at T_{IMT} drives the system into the metallic state and to a lower ρ. Now, to reach the new transition temperature for the non-zero doping state, the temperature has to be lowered below T_{IMT}. Since $d\rho/dT$ is positive in the metallic state, the resistance at the transition point for $\delta>0$ has to be lower than $\rho(T_{IMT},\delta=0)$.

The critical doping levels obtained from our DMFT calculations depend on T. In the range $T/T_{IMT} < 0.75$ relevant to our experiments, the critical doping ranges from 0 to 10%. For example, in cuprates, doping levels of ~5% are required for suppression of the Mott insulator in reasonable agreement with our calculations. For V_2O_3, consistent with our simple model, there is also a critical doping dependence with T. However, there is a quantitative difference, since smaller levels of doping destabilize the Mott insulator state, as reported in several studies of $V_{2(1-y)}O_3$ which showed that vanadium deficiency on the order of $\lesssim 1\%$ suppresses the transition.[16,17] Our model is very simple, and despite the differences in critical doping value, the qualitative behavior is consistent.

We can roughly estimate the carrier doping at the switching point in our devices from the irradiation dose and field enhancement of the conductivity. In irradiated samples the ion dose is known so that the number of induced defects can be estimated. SRIM (stopping range of ions in matter) simulations give an average of 450 vacancies per Ga ion (30 keV).[18] For a dose of 6.2×10^{14} ion cm^{-2} (see irradiated V_2O_3 nanowire discussed in Fig. 3(c) and (d) of the main text) this translates into ~3 vacancies per unit cell, which includes 30 V and O atoms. Given the activation energy of this sample (~50 meV) the fraction of thermally ionized

defect states for zero applied voltage is around 0.12% at 85 K, which is not large enough to suppress the IMT. However, at the switching voltage of 10.3 V (~2.9 MV/m), the fraction of ionized defects increases to 9.5% as we find from a fit to Poole-Frenkel conductivity enhancement (see Supplementary Fig 7(a)). Given the estimated defect density, this result is in agreement with critical doping levels of ~1% found in previous studies. We note that for the irradiated VO_2 nanowire discussed above (section 5) the conductivity enhancement does not follow the Poole-Frenkel formula. As shown in Supplementary Fig. 7(b), instead of $\ln(\sigma) \propto V^{0.5}$ the conductivity follows $\ln(\sigma) \propto V^2$, indicative of phonon assisted tunneling.[19] This may be related to the higher relative permittivity of VO_2 (ε_r ~ 36)[20] compared to V_2O_3 (ε_r = 5-17 – see Supplementary Fig. 7)[21] and the higher measurement temperature for VO_2. The combined effect of these parameters is to reduce the efficiency of the Poole-Frenkel effect in VO_2 by a factor of ~10 compared to V_2O_3. Additionally, the shape and magnitude of the trapping potential affects the efficiency of field assisted carrier excitation. We note that the activation energy in VO_2 is up to ~5 times larger than in V_2O_3, as shown in section 3. This may leave phonon assisted tunneling as the dominant contribution to field assisted excitation of carriers. The relatively low efficiency of carrier excitation in VO_2 could be the reason for the higher irradiation doses and fields required to achieve switching compared to V_2O_3. This is a subject of future work.

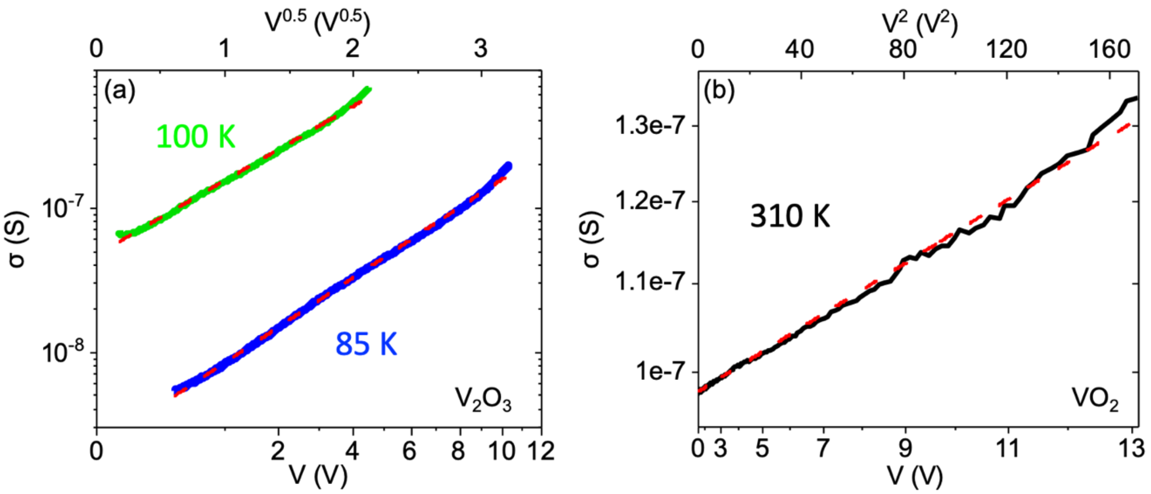

Supplementary Fig. 7: Field assisted carrier generation in irradiated VO_x films. (a) Fit to Poole-Frenkel conductivity enhancement (before switching) for the irradiated V_2O_3 nanowire (6.2×10^{14} ions cm^{-2}) discussed in Fig. 3(c) and (d) of the main text, at different temperatures as indicated. Red dashed lines are fits to $\sigma = \sigma_0 \exp\left[\frac{1}{k_B T}\left(\frac{e^3 V}{\pi\varepsilon_r\varepsilon_0 d}\right)^{0.5}\right]$ - k_B is the Boltzmann constant, ε_0 is the vacuum permittivity and d=3.5 μm is the nanowire length. The relative permittivities extracted from the fits are ε_r=16.8 (85 K) and ε_r=15.9 (100 K). These values are between those derived from optical measurements on V_2O_3 (~5)[21] and those of insulating VO_2 at 1 MHz (~36).[20] (b) Fit to conductivity enhancement due to phonon assisted tunneling for the irradiated VO_2 nanowire (6.2×10^{15} ions/cm^2) discussed in Supplementary Fig. 4. Red dashed line is a fit to $\sigma = \sigma_0 \exp\left[\left(\frac{V/d}{E_0}\right)^2\right]$, with d=3.5 μm and the extracted E_0=6.9 MV/m.

Supplementary Note 7: Switching energy measurements

In this section we discuss the switching energy of the nanowire shown in Fig. 2(c,d) in the main text. The energy was derived from the integrated power applied to the sample during a voltage pulse. Pulses were applied in the fully insulating state so that power is uniformly distributed along the nanowire. To observe a change in resistance after switching, the sample had to be in the coexistence regime where some metallic domains would remain stable after applying the pulse. Before applying pulses, V(I) measurements were performed at T_0=112 K from which V_{switch}=5.5 V was determined (supplementary Fig. S9). Due to hysteresis, the resistance of the sample after switching was lower than in the initial state (Fig. S8). We used this resistive hysteretic behavior as an indicator for whether switching had occurred in response to the voltage pulse. The resistance was measured in the DC limit by applying a continuous current of 10 nA while 6 ns voltage pulses of varying amplitudes were applied using a bias tee (see also methods). This is an excellent alternative to dynamical resistance measurements which are hindered by the high impedance of our samples (10^7-10^8 Ω). It provides a simple way of identifying the first switching event and estimating the incubation time and energy associated with it.

Twenty pulses were applied with amplitudes of 4.5 V and 4.9 V with no observable change in sample resistance. Next, a single 5.3 V pulse was applied and switching of $\Delta R/R$~24% was observed, consistent with the DC measurements showing V_{switch}=5.5 V and a similar change in resistance (~31%) (see Fig. S9).

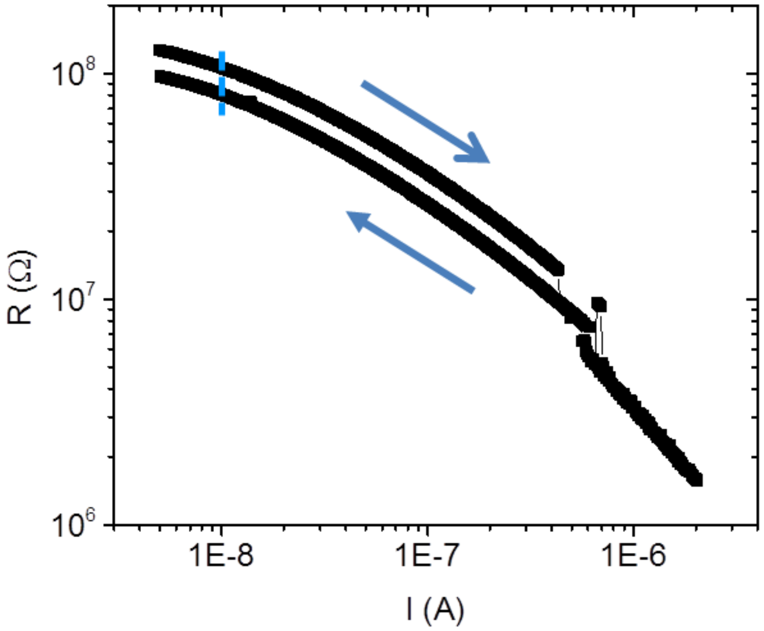

Supplementary Fig. S8: (a) R(I) curve acquired at 112 K corresponding to the R(V) measured in Fig. S9. Since the sample is in the coexistence regime some metallic domains persist after the current is ramped down resulting in a hysteretic R(I) curve. The dashed line corresponds to the small probe current used to detect a change in resistance due applied pulses in Fig. S9.

Using the known *V*(t) during the pulse and steady state *R(V)* curve we derived the power input to the nanowire as a function of time:

$$P(t) = \frac{V(t)^2}{R\big(V(t)\big)}$$

By integrating the power over the duration of the pulse, the total energy delivered to the nanowire was found to be 5 fJ. Using the literature value for the heat capacitance of V_2O_3 (~35 J/mol·K)[9], this corresponds to a ~120 mK increase in the nanowire temperature. Since the resistance change for such a small temperature increase is extremely small, no thermal instabilities are expected to occur in this

case. This supports the thermal stability simulations and rules out thermal runaway effects in the insulating state, as suggested in a previous study.[7] We note that the shortest pulse duration we could apply was 6 ns, so that 5 fJ is an upper bound for the energy required to induce the transition. In fact, IMT transition times as fast as several picoseconds have been observed in pump-probe experiments for V_2O_3.[22,23] If this transition could be electrically triggered with a similar voltage over picoseconds instead of nanoseconds, the switching energy may be reduced by at least two orders of magnitude.

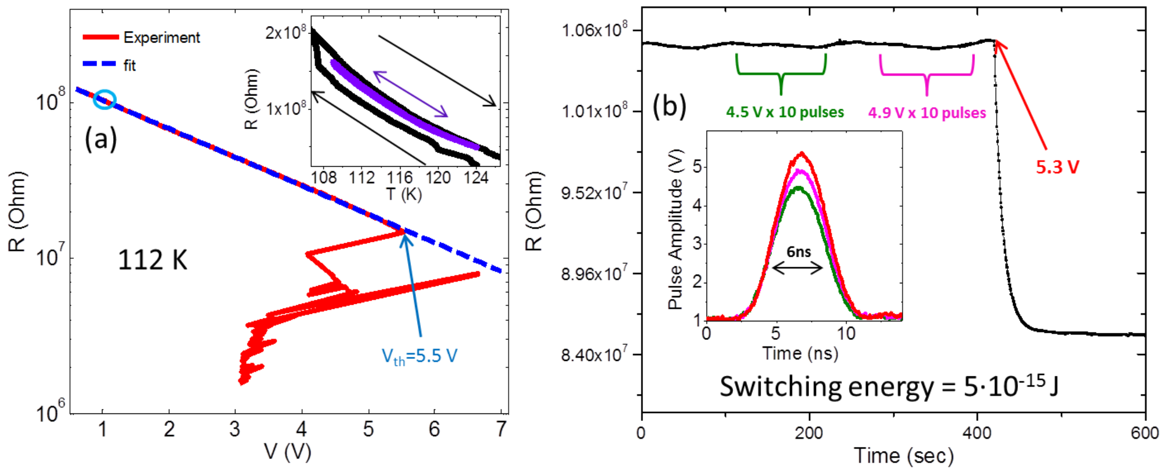

Supplementary Fig. S9: (a) R(V) curve acquired at 112 K showing switching at 5.5 V. This curve is used to determine the resistance during the voltage pulse shown in (b). The inset to (a) shows $R(T)_{eq}$ curves acquired along the full IMT loop (black) and along a minor loop (purple curve - 109 K → 124 K → 109 K). The minor loop shows no hysteresis. (b) R(time) acquired with I=10 nA (equivalent to light blue circle in (a) at ~1 V) while 6 ns voltage pulses with varying amplitudes are applied to the sample. The various pulses are shown in the inset. 20 pulses of 4.5 V and 4.9 V did not show any resistance change while the first 5.3 V pulse triggered a large

change in resistance, in accordance with the threshold voltage measured in (b). The slow relaxation of resistance observed after switching is due to RC discharge.

To compare the switching energy to what is expected from a Joule-heating driven transition we measured the lowest temperature required to observe an IMT in part of the sample in equilibrium. We performed an R(T) minor loop following the sequence 109 K → 124 K → 109 K (see purple curve in inset to Fig. S9(a)). No jumps were observed during this measurement and the sweeps up and down in temperature were repeatable. We thus conclude that for a Joule heating scenario, the sample must be heated from T_0=112 K to a temperature above 124 K to induce the IMT in a portion of the nanowire. Based on the heat capacitance, we find that over ~1.5 pJ must be imparted to the nanowire to thermally drive into IMT. This value would be even larger if heat dissipation during the pulse were also considered. Moreover, the latent heat associated with the transition for the entire nanowire is ~7 pJ. Thus, the thermal switching energy is about three orders of magnitude larger than the energy delivered to the nanowire during the pulse (~5 fJ), indicating a negligible role played by Joule heating. To impart several pJ via a 6 ns voltage pulse, the resistance of the sample should be less than 10 kΩ. Since this resistance is on the order of the metallic state resistance (4 kΩ), it is impossible for the pulse to deliver the required thermal switching energy while the sample is in the insulating state. This further corroborates the implausibility of thermal runaway effects as the mechanism behind the observed electrical switching.

These switching characteristics are in stark contrast to the case of the VO_2 nanowire discussed in Fig. 2(a,b) of the main text. For example, at 334 K, almost 5 times higher voltage (25 V) and 75 times larger power (~150 μW equivalent to

ΔT~3.5 K) can be continuously applied without switching the VO_2 nanowire, despite being very close to the IMT.

Suppplementary References

1. Sharoni, A., Ramírez, J. G. & Schuller, I. K. Multiple Avalanches across the Metal-Insulator Transition of Vanadium Oxide Nanoscaled Junctions. *Phys. Rev. Lett.* **101**, 26404 (2008).

2. McLeod, A. S. *et al.* Nanotextured phase coexistence in the correlated insulator V_2O_3. *Nat. Phys.* **13**, 80 (2016).

3. Wu, J. *et al.* Strain-Induced Self Organization of Metal−Insulator Domains in Single-Crystalline VO2 Nanobeams. *Nano Lett.* **6**, 2313–2317 (2006).

4. del Valle, J. *et al.* Resistive asymmetry due to spatial confinement in first-order phase transitions. *Phys. Rev. B* **98**, 45123 (2018).

5. Madan, H., Jerry, M., Pogrebnyakov, A., Mayer, T. & Datta, S. Quantitative Mapping of Phase Coexistence in Mott-Peierls Insulator during Electronic and Thermally Driven Phase Transition. *ACS Nano* **9**, 2009–2017 (2015).

6. Guénon, S. *et al.* Electrical breakdown in a V_2O_3 device at the insulator-to-metal transition. *Europhys. Lett.* **101**, 57003 (2013).

7. Brockman, J. S. *et al.* Subnanosecond incubation times for electric-field-induced metallization of a correlated electron oxide. *Nat. Nanotechnol.* **9**, 453 (2014).

8. Kumar, S. *et al.* Physical origins of current and temperature controlled negative differential resistances in NbO2. *Nat. Commun.* **8**, 658 (2017).

9. Lyakh, O. V, Surikov, V. I., Surikov, V. I. & Prokudina, N. A. Magnetic susceptibility and heat capacity of V_2O_3 and $V_{1.973}Me_{0.020}0O_3$ (Me = Fe, Cr, and Al). *Russ. Phys. J.* **55**, 116–119 (2012).

10. Valmianski, I. *et al.* Origin of the current-driven breakdown in vanadium oxides: Thermal versus electronic. *Phys. Rev. B* **98**, 195144 (2018).

11. Georges, A., Kotliar, G., Krauth, W. & Rozenberg, M. J. Dynamical mean-field theory of strongly correlated fermion systems and the limit of infinite dimensions. *Rev. Mod. Phys.* **68**, 13–125 (1996).

12. Gull, E. *et al.* Continuous-time Monte Carlo methods for quantum impurity models. *Rev. Mod. Phys.* **83**, 349–404 (2011).

13. Mazza, G., Amaricci, A., Capone, M. & Fabrizio, M. Electronic transport and dynamics in correlated heterostructures. *Phys. Rev. B* **91**, 195124 (2015).

14. Mazza, G., Amaricci, A., Capone, M. & Fabrizio, M. Field-Driven Mott Gap Collapse and Resistive Switch in Correlated Insulators. *Phys. Rev. Lett.* **117**, 176401 (2016).

15. Kalcheim, Y. *et al.* Robust Coupling between Structural and Electronic Transitions in a Mott Material. *Phys. Rev. Lett.* **122**, 57601 (2019).

16. Shivashankar, S. A. & Honig, J. M. Metal-antiferromagnetic-insulator transition in V2O3 alloys. *Phys. Rev. B* **28**, 5695–5701 (1983).

17. Carter, S. A., Rosenbaum, T. F., Honig, J. M. & Spalek, J. New phase boundary in highly correlated, barely metallic V2O3. *Phys. Rev. Lett.* **67**, 3440–3443 (1991).

18. Biersack, J. P. & Ziegler, J. F. The Stopping and Range of Ions in Solids BT - Ion Implantation Techniques. in (eds. Ryssel, H. & Glawischnig, H.) 122–156 (Springer Berlin Heidelberg, 1982).

19. Ganichev, S. D. *et al.* Distinction between the Poole-Frenkel and tunneling models of electric-field-stimulated carrier emission from deep levels in semiconductors. *Phys. Rev. B* **61**, 10361–10365 (2000).

20. Yang, Z., Ko, C., Balakrishnan, V., Gopalakrishnan, G. & Ramanathan, S. Dielectric and carrier transport properties of vanadium dioxide thin films across the phase transition utilizing gated capacitor devices. *Phys. Rev. B* **82**, 205101 (2010).

21. Qazilbash, M. M. *et al.* Electrodynamics of the vanadium oxides VO2 and V2O3. *Phys. Rev. B* **77**, 115121 (2008).

22. Singer, A. *et al.* Nonequilibrium Phase Precursors during a Photoexcited Insulator-to-Metal Transition in V_2O_3. *Phys. Rev. Lett.* **120**, 207601 (2018).

23. Abreu, E. *et al.* Dynamic conductivity scaling in photoexcited V_2O_3 thin films. *Phys. Rev. B* **92**, 85130 (2015).
